# The application of artificial intelligence in the acute and sub-acute phases of spinal cord injury- a systematic review

**DOI:** 10.1038/s41393-025-01155-0

**Published:** 2025-12-04

**Authors:** Teleale F. Gebeyehu, Mohammad Amin Sabbaghalvani, Giovanna Failla, Ashmal S. Kabani, Yashvi Shah, Alexander Kharichev, Joshua A. Dian, Stavros Matsoukas, Alexander R. Vaccaro, Gregory D. Schroeder, Srinivas K. Prasad, Jack Jallo, Joshua E. Heller, Michael G. Fehlings, James S. Harrop

**Affiliations:** 1 Department of Neurological Surgery, Thomas Jefferson University, Philadelphia, PA 19107 US; 2 Department of Neurological Surgery, Shohada Tajrish Hospital, Shahid Beheshti University of Medical Sciences, Tehran, Iran; 3 Università Cattolica del Sacro Cuore, Graduate School of Health Economics and Management (ALTEMS), Largo Francesco Vito, 1, 00168 Roma, Italy; 4 Drexel University, College of Medicine, Philadelphia, PA 19102 US; 5 Department of Neurosurgery, Ankara University Medical Faculty, Emniyet, Dögol Cd., 0600 Ankara, Türkiye; 6 Rothman Orthopedics Institute, Philadelphia, PA 19107 US; 7 University of Toronto, Division of Neurosurgery and Spine Program, Toronto, M5T 2S8 ON Canada

**Keywords:** Health services, Diagnosis, Prognosis, Quality of life

## Abstract

**Study design:**

Systematic Review.

**Objective:**

To describe applications of AI for traumatic SCI management with focus on diagnostics, prognostication, and therapeutic interventions.

**Methods:**

PubMed, Scopus and Cochrane libraries were searched (March 2025). Studies published in English between January 1^st^, 2020, and March 18, 2025, dealing with clinical aspects in the acute, post-injury rehabilitative and first year phases of SCI were included. Studies on brain computer interface, robotics and non-neurologic aspects of SCI were excluded. Extracted were country of study, study design, focus of study, total participants, American Spinal Injury Association (ASIA) Impairment Scale (AIS), machine learning (ML) models, inputs, outcomes and performance metrices.

**Results:**

A total of 23 studies with 120,931 individuals were identified. Classical Machine Learning Models, Ensemble Learning Models and Deep Learning Models were the most used ML families. Age, AIS, neurologic level of injury, sex, mechanism of injury and motor score were the most common inputs. Predictions of neurologic status, functionality status, Hospital/ICU utilizations, complications, survival, discharge destination and results of image segmentation and patient grouping were the outputs of interest. The performance metrices were satisfactory in most and higher than humans in some studies.

**Conclusion:**

AI can facilitate personalized approach to diagnosis of SCI, prediction of outcomes like neurological improvement, complications, functionality indicators like walking, selfcare and independence, re-admissions, prolonged length of stays, discharge destination and mortality after injury. It was also useful to suggest specific MAP goals and time of surgical intervention. These functions complement clinical judgement.

## Introduction

Spinal cord injury (SCI) is a life-altering event that will cause substantial neurological impairment, such as Loss of sensory, motor, and autonomic functions, leaving a significant burden on the healthcare system. Approximately 15.4 million individuals around the globe are living with this problem [1, 2]. Moreover, incidence rates of traumatic SCIs, which account for more than 90% of all SCIs, are up to 906 cases per million globally. Traumatic SCI is most often due to road traffic accidents, falls, or violence, with young adults and older adults being the most affected groups [3–5]. Addressing this challenge has been a priority among health care professionals, and for better outcomes, the emphasis has been on cross-specialty collaboration and time-intensive actions. SCI is a complex and heterogeneous phenomenon in terms of injury mechanism, level, and severity. Thus, traditional approaches often fail to address the diverse needs of Individuals with SCI for timely diagnosis, accurate prognostication, and therapeutic decision-making [6, 7].

Artificial intelligence (AI) has emerged as a promising new tool for enhancing clinical care through automated data analysis, pattern recognition, and predictive modeling. AI, with the use of predictive analytics, machine learning (ML), and deep learning (DL), has shown promising results in enhancing clinical decision-making and personalizing treatment strategies for Individuals with SCI [8, 9]. Advanced ML models, such as convolutional neural networks and support vector machines (SVM), have improved the accuracy of neuroimaging analysis, enabling precise identification of injury severity through automated segmentation of MRI and CT scans. These advancements can help answer one of the most important questions regarding traumatic SCIs: will a given patient benefit from prompt surgical intervention or not? [10, 11] In prognostication studies, ML algorithms have demonstrated that they can predict neurological outcomes, such as ambulatory ability and neurological recovery following SCI, based on acute clinical and imaging data with moderate predictive performance [12, 13].

Despite all the benefits and growing interest, the integration of AI into routine clinical workflows for SCI remains limited. Issues such as lack of external validation, small sample sizes, lack of interpretability, and barriers continue to hinder clinical adoption. In this systematic review, we aim to detail the current evidence on the application of AI in the clinical management of traumatic SCI, with a focus on its efficacy in diagnostics, prognostication, and therapeutic interventions. In turn, this review seeks to provide clinicians and researchers with a comprehensive understanding of AI’s transformative potential in SCI care and address the gaps in clinical integration, thus guiding the development of standardized AI tools and improving patient outcomes.

## Methods

In this PRISMA-2020 compliant review, Medline (PubMed) was queried using the MeSH term “((artificial intelligence) OR (machine learning)) AND (traumatic spinal cord injury)” while Scopus and Cochrane libraries were searched using the keywords in the MeSH term on March 18, 2025, at 11:53 am (TG). Citations within the included studies were manually checked on the study subject (TG, ASK, YS, GF, AK). Studies published in English between January 1st, 2020, and March 18, 2025, dealing with clinical aspects in the acute, post-injury rehabilitative, and first-year phases of SCI were included. We excluded studies focusing on brain computer interfaces, robotics for limb function, and non-neurologic aspects of SCI. One author (TG) screened the titles and abstracts, and five authors (TG, ASK, YS, GF, AK) conducted a full screen independently, extracting data and deciding whether to include or exclude studies. Disagreements were resolved with discussion. Extracted data included country of study, study design (prospective, retrospective, clinical trial etc.), focus of the study (diagnosis [image segmentation], prediction of outcomes, patient categorization and management suggestions), total number of patients, the American Spinal Injury Association (ASIA) Impairment Scale (AIS), the ML model/AI used, variables used by the ML model, outputs, and performance metrices (sensitivity/recall, specificity, positive predictive value, negative predictive value, area under receiver operating characteristic curve, area under precision recall curve, mean absolute error, mean squared error, mean absolute percentage error, root mean squared error, accuracy, precision, dice score, interaction over union, relative volume error, surface distance, Matthews correlation coefficient, kappa coefficient, F1 score, Brier score and training time).

Calculation of the total percentage of ML types was based on the number of uses in the study, since each ML subtype can evaluate different outcomes. To better classify and understand the focus of AI utilization for Individuals with SCI, four main categories of focus were created: Diagnosis or Image segmentation, Patient Categorization, Prediction of Outcome, and Prediction of Outcome with Management Suggestion. This categorization was dependent on the specific contents of the study. Similarly, the outcomes generated were grouped into eight main clinical categories based on the similarity of the clinical outcomes assessed (Complications, Discharge Destination, Functionality Status, Hospital/ICU Utilization, Image Segmentation, Neurologic Status, Patient Grouping, and Survival). ChatGPT (OpenAI) [14] was utilized to identify subtypes and families of the ML models. Due to the heterogeneity of the ML model used, outcomes, and performance metrics across studies, a meta-analysis was not possible to conduct. However, qualitative data were extracted from the presented results. Every specific ML model used, and the performance measures are reported in a tabular format for a subjective and qualitative review (Supplement Materials). Two authors (TG and GF) independently performed quality and ROB assessment. Disagreements were settled with discussion. Quality of model development and Risk of bias (ROB) for the predictive models was assessed using the newly developed Prediction Model Risk of Bias Assessment Tool for Artificial Intelligence (PROBAST + AI) [15]. Quality of development and ROB for diagnostic models was assessed utilizing the domains of quality assessment of diagnostic accuracy studies (QUADAS-2) tool [16] with a custom modification based on PROBAST + AI and another study suggesting areas of potential bias for AI based diagnostic tools [17]. Study characteristics are displayed in a tabular format. This study was registered in PROSPERO (CRD420251021149) and a concise purpose, methods, and rationale of this study can be found there. All calculations and visualizations were created using Microsoft® Excel® for Microsoft 365 MSO (Version 2503 Build 16.0.18623.20178) 64-bit.

## Results

**Fig. 1:**
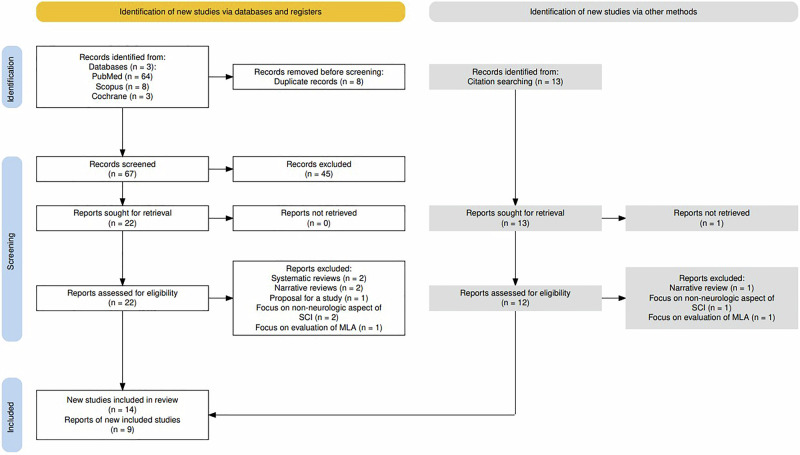
- Prisma Flow Diagram [18] for study selection. MLA- Machine Learning Algorithm, SCI- Spinal Cord Injury.

The PRISMA flow diagram [18] of the literature selection process is displayed in (Fig. 1). Initial search resulted in 88 studies (64 in PubMed, 8 in Scopus, 3 in Cochrane, and 13 from citations), 8 of which were duplicates. The studies identified from the databases were screened using titles and abstracts, resulting in 22 studies that were selected and retrieved for full review. All 12 studies from the citations, except for one unretrieved study, were thoroughly reviewed. After the full review, 2 systematic reviews [13, 19], 3 narrative reviews [10, 20, 21], 1 proposal for a study [22], 2 studies of machine learning algorithm (MLA) evaluation [23, 24] and 3 studies focusing on non-neurologic aspects of SCI [25–27] were excluded, resulting in a total of 23 studies [12, 28–49] to be included in the final review. One of the excluded studies [50] was based on the clinical information from a study already included [28] and shared similar authors.

Quality and ROB assessment results are shown in Table 1. For the development of models, 16/22 studies had high and, 6/22 low concern for quality. One study had unclear quality of development. Again, for the development of the models, applicability concern was low for 20/23 studies, high for 2/23 studies and one study had unclear applicability. For model validation, 17/22 had high, 4/22 low and 1/22 unclear concern for ROB. Applicability wise, model validation was of low concern for 20/22 studies while 2/22 studies were of high concern. One study [30] was on development SCI patient’s classification and is neither diagnostic or predictive model with no validation or testing. For this reason, it was not feasible to apply the tools we used to assess quality and ROB. However, it is applicable to the review and is included.

**Table 1:** Quality and Applicability assessment of machine learning model development and validation in the included studies.

Author, Year	Model Development									Model Validation								
	Participants and Data Source		Predictors		Outcome		Analysis	Overall		Participants and Data Source		Predictors		Outcome		Analysis	Overall	
	Quality	Applicability	Quality	Applicability	Quality	Applicability	Quality	Quality	Applicability	Risk of Bias	Applicability	Risk of Bias	Applicability	Risk of Bias	Applicability	Risk of Bias	Risk of Bias	Applicability
**Predictive Studies (PROBAST** + **AI)**																		
Agarwal et al. [28]	Low	Low	Low	Low	High	Low	High	High	Low	Low	Low	Low	Low	Low	Low	High	High	Low
Aly et al. [29]	Low	Low	Low	Low	Low	Low	Low	Low	Low	Low	Low	Low	Low	Low	Low	High	High	Low
Basiratzadeh et al. [30]	N/A	N/A	N/A	N/A	N/A	N/A	N/A	N/A	N/A	N/A	N/A	N/A	N/A	N/A	N/A	N/A	N/A	N/A
Bruningk et al. [31]	Low	Low	Low	Low	Low	Low	High	Low	Low	Low	Low	Low	Low	Low	Low	Low	Low	Low
De Vries et al. [32]	Low	Low	Low	Low	Low	Low	High	High	Low	Low	Low	Low	Low	Low	Low	Unclear	Unclear	Low
Facchinello et al. [12]	High	Low	High	Low	High	Low	High	High	Low	Low	Low	Low	Low	Low	Low	High	High	Low
Fallah et al. [33]	Low	Low	Low	Low	Low	Low	High	High	Low	Low	Low	Low	Low	Low	Low	Low	Low	Low
Fan et al. [34]	High	Low	Low	Low	Low	Low	High	High	Low	Low	Low	Low	Low	Low	Low	High	High	Low
Goulet et al. [35]	Low	Low	Low	Low	Low	Low	High	High	Low	Low	Low	High	Low	Low	Low	High	High	Low
Inoue et al. [36]	High	Low	Low	Low	Low	Low	High	High	Low	High	Low	Low	Low	Low	Low	High	High	Low
Kapoor et al. [37]	Low	Low	Low	Low	Low	Low	High	High	Low	Low	Low	Low	Low	Low	Low	High	High	Low
Karaback et al. [38]	High	High	Low	Low	High	Low	Low	High	High	High	High	Low	Low	High	Low	High	High	High
Kato et al. [39]	Low	Low	Low	Low	Low	Low	High	High	Low	Low	Low	Low	Low	Low	Low	High	High	Low
Kitagawa et al. [40]	Low	Low	Low	Low	Low	Low	High	High	Low	Low	Low	Low	Low	Low	Low	High	High	Low
Maki et al. [41]	Low	Low	Low	Low	Low	Low	Low	Low	Low	Low	Low	Low	Low	Low	Low	Low	Low	Low
Okimatsu et al. [43]	Low	Low	Low	Low	Low	Low	Low	Low	Low	Low	Low	Low	Low	Low	Low	High	Low	Low
Shimzu et al. [45]	Low	Low	Low	Low	Low	Low	High	High	Low	Low	Low	Low	Low	Low	Low	High	High	Low
Torres-Espin et al. [46]	Low	Low	Low	Low	Low	Low	Low	Low	Low	Low	Low	Low	Low	Low	Low	High	High	Low
Yoo et al. [47]	High	Low	Low	Low	Low	Low	High	High	Low	High	Low	Low	Low	Low	Low	High	High	Low
Zhang et al. [48]	Low	Low	Low	Low	Low	Low	High	High	Low	Low	Low	Low	Low	Low	Low	High	High	Low
	**Model Development**									**Model Validation**								
**Diagnostic Studies (QUADAS AI Based)**	**Participants and Data Source**		**Index Test**		**Reference Standard**		**Flow and Timing**	**Overall**		**Participants and Data Source**		**Index Test**		**Reference Standard**		**Flow and Timing**	**Overall**	
	**Quality**	**Applicability**	**Quality**	**Applicability**	**Quality**	**Applicability**	**Quality**	**Quality**	**Applicability**	**Risk of Bias**	**Applicability**	**Risk of Bias**	**Applicability**	**Risk of Bias**	**Applicability**	**Risk of Bias**	**Risk of Bias**	**Applicability**
Artha Wiguna et al. [49]	High	Low	Unclear	Low	Low	Low	N/A	High	Low	High	Low	Unclear	Low	Low	Low	Unclear	High	Low
Naga Karthik et al. [42]	Low	Low	Low	Low	Low	Low	N/A	Low	Low	High	Low	Low	Low	Low	Low	Low	High	Low
Sharafi et al. [44]	High	Low	High	Low	Low	Low	N/A	High	Low	High	Low	High	Low	Low	Low	Low	High	Low

Of the 23 studies we included, 6 were conducted in the USA, 6 in Japan, 5 in Canada, 2 in China, 1 in Indonesia, 1 in Korea, and 2 were results of work conducted in multiple countries, namely France, Germany, Switzerland, Canada, and the USA. Two of the 23 studies were prospective, while the rest were retrospective. There were 120,637 individuals whose various data were used for machine learning, validation, and testing. A total of 15 of the 23 studies reported the AIS scale for patients (n = 12,599). The most common AIS score was A (4463; 35.4%), followed by D (3857; 30.6%), C (2868; 22.8%), and B (1376; 10.9%). Table 2 provides a summary of these findings.

**Table 2:** Summary of study characteristics, including authors, country, study design, total number of patients, count or percentage of patients with a given American Spinal Injury [ASIA] Impairment Scale (AIS) score, and Focus of the Study.

Author, Year	Country	Study Design	Total Number of Patients/Images	ASIA Score (A, B, C, D & E)	Focus of Study	Outcome Assessed
Agarwal et al. [28]	USA	Retrospective	74	A = 37, B = 8, C = 14, D = 13, E = 2	Management and Prediction of outcome	AIS score at discharge and Cardiovascular Complication. Ideal MAP range (76 – 104 mmHg)
Aly et al. [29]	USA	Retrospective	4932	N/A	Prediction of outcome	Re-hospitalization and prolonged length of stay after re-admission during the first year after SCI.
Artha Wiguna et al. [49]	Indonesia	Retrospective	N/A	N/A	Diagnosis (Image Segmentation)	Sagittal and axial spinal cord segmentation. Classify SCI severity.
Basiratzadeh et al. [30]	Canada	Retrospective	334	A = 125, B = 48, C = 62, D = 99	Patient Categorization	SCI patient Subgroups.
Brüningk et al. [31]	Mutiple (Switzerland, Germany, Canada, USA)	Retrospective	1678	A = 815, B = 224, C = 316, D = 323	Prediction of outcome	Segmental Motor/Sensory score, walking, AIS conversion (at least one level) at and after six months of SCI.
DeVries et al. [32]	Canada	Retrospective	862	A = 257, B = 79, C = 149, D = 379	Prediction of outcome	Walking Ability at one year and thereafter of SCI.
Facchinello et al [12]	Canada	Prospective	172	A = 39.5%, B = 9.9%, C = 14.5%, D = 36.1%	Prediction of outcome	SCIM score using four and eleven predictors at one year or six-months after SCI.
Fallah et al. [33]	Canada	Retrospective	1245	A = 461, B = 117, C = 227, D = 400	Prediction of outcome	In-hospital, one-year, and seven-year mortality using SCIRS developed by ML.
Fan et al. [34]	China	Retrospective	1599	N/A	Prediction of outcome	Length of Stay in hospital and ICU.
Goulet et al. [35]	Canada	Prospective	35	A = 28, B = 7	Management and Prediction of outcome	AIS, NLI, and motor score at one year of SCI. Timing of surgery after SCI.
Inoue et al. [36]	Japan	Retrospective	165	N/A	Prediction of outcome	Neurological Recovery at six months after injury based on AIS score (Dichotomized into AIS A/B/C Group or D/E group)
Kapoor et al. [37]	USA	Retrospective	20,790	N/A	Prediction of outcome	AIS at discharge.
Karabacak et al. [38]	USA	Retrospective	79,769	N/A	Prediction of outcome	In-hospital Mortality, Non-Home Discharges, Prolonged LOS, Prolonged ICU-LOS, Major Complications.
Kato et al. [39]	Japan	Retrospective	210	A = 47, B = 30, C = 44, D = 89	Prediction of outcome	Discharge Destination (Home/not home).
Kitagawa et al. [40]	Japan	Retrospective	3703	A = 1217, B = 393, C = 928, D = 1047	Prediction of outcome	AIS at discharge.
Maki et al. [41]	Japan	Retrospective	3827	A = 1266, B = 386, C = 921, D = 1052	Prediction of outcome	Achievement of Functional Ambulation and total FIM score at discharge from Rehab.
Naga Karthik et al. [42]	Multiple (France, Switzerland and USA)	Retrospective	191	N/A	Diagnosis (Image Segmentation)	Spinal Cord and Lesion segmentation.
Okimatsu et al. [43]	Japan	Retrospective	215	A = 25, B = 19, C = 62, D = 82, E = 27	Prediction of outcome	AIS at one month after SCI.
Sharafi et al. [44]	USA	Retrospective	62	A = 2, B = 2, C = 3, D = 5	Diagnosis (Image Segmentation)	Discrimination between Controls and Individuals with SCI, injury severity categorization, and lesion zone classification using T1W and T2W MRI.
Shimzu et al. [45]	Japan	Retrospective	135	A = 35, B = 17, C = 48, D = 35, E= Excluded	Prediction of outcome	Neurological outcomes six months after SCI, binary or 5 class based on AIS score.
Torres-Espin et al. [46]	USA	Retrospective	118	A = 52, B = 13, C = 16, D = 19, E = 4, Missing= 15	Management and Prediction of outcome	AIS Improvement, AIS A and D at Discharge. Intra-op MAP range (76 – 104/117 mmHg).
Yoo et al. [47]	Korea	Retrospective	353	A = 29, B = 18, C = 53, D = 251, E = 2	Prediction of outcome	Functional Ambulation Category at Discharge.
Zhang et al. [48]	China	Retrospective	168	N/A	Prediction of outcome	International Association of Neurorestoratology (IANR) score six months after SCI.
						SCI Severity Classification based on BASIC scores- 5 Class (scores 0 - 4) or 3 Class- (**Class 0**- scores 0 and 1, **Class 1**- Score 2, **Class 3**- Scores 3 and 4)

N/A- Not Available.

All the AI types and their specific performance metrics are shown in Supplementary Material S1. Supplementary material S2 summarizes the percentages for the families and subtypes of MLAs according to the classification by OpenAI [14]. The AI/ML models used in these studies are mostly of the Classical Machine Learning Model family (47.5%), followed by the Ensemble Learning Model family (34.6%), and the Deep Learning Model family (13%). Among the classical machine learning models, the subtypes, linear models (linear and logistic regression, among others) comprised 17.3% and tree-based models (decision tree, random forest and regression tree), each comprised 16%, while Nearest Neighbor model (commonly known as k-NN) and support vector machine (SVM) comprised 8% of the total subtypes. Ensemble models, which are part of the Ensemble Learning Model family, comprise 28.4% of the total number of subtypes and included Adaptive Boosting (AdaBoost, 4.9%), Categorization Boosting (CatBoost, 5.6%), Gradient Boost (GB, 3.1%), Light Gradient Boosting Machine (LightGBM, 6.8%), and Extreme Gradient Boosting Machine (XGBoost, 8%). Convolutional Neural Networks (CNN) were the most common of the deep learning models, constituting 9.9% of the ML models used. While the three mentioned families of ML made 95.1%, the rest were constituted by the AutoML-Generated Model (1.9%), Hybrid/Combined Model (1.2%), and Unsupervised Learning Model (1.9%), which can constitute multiple subtypes mixed from the earlier families depending on the data and the designer’s choices [14].

**Fig. 2:**
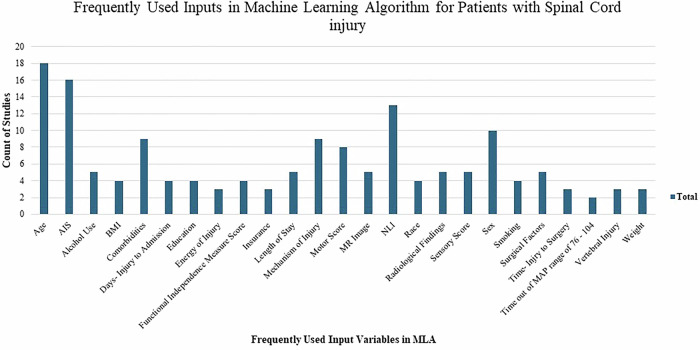
Commonly used inputs in machine learning algorithms for spinal cord injury application. - AIS- American Spinal Injury Association [ASIA] Impairment Scale, BMI- Body Mass Index, MAP- Mean Arterial Pressure, MR- Magnetic Resonance, NLI- Neurologic Level of Injury.

Different studies used differing inputs, so summarizing them all was not feasible given the vast variation. In most studies, Shapley additive explanations (SHAP) identified the most influential inputs. Figure 2 shows the commonly used inputs, with age, AIS, NLI, sex, mechanism of injury, and motor score comprising the top inputs in various studies. Other notable inputs to mention are injury severity score, smoking, vertebral fracture, vital signs, presence of traumatic brain injury, Charleson comorbidity index, sequential motor and sensory scores, anal sensation, bladder management, and Brain and Spinal Injury Center (BASIC) score.

Table 2 summarizes the specific outcomes the studies evaluated using MLAs. Supplementary material S3 stratifies the studies under the study’s specific focus and category of outcomes. The most common study focus was the use of AI/ML for predicting outcomes in Individuals with SCI (17/23 studies), while the rest focused on AI/ML use for diagnosis/image segmentation to detect pathology using MRI (3/23). Others focused on the combined prediction of outcome and management suggestions (3/23) and patient categorization (1/23). Evaluating the studies closely revealed there were 8 major categories of outcomes that were studied using ML algorithms. The most common outcome was neurological status, which was described in 10/23 studies, followed by functionality status in 6/23, Image segmentation results in 3/23, Hospital/ICU utilizations in 3/23, Complications in 2/23, survival in 2/23, discharge destination in 2/23, and results of patient grouping in 1/23. More specifically, neurologic status was assessed using AIS changes at discharge, 1 month, 6 months, and 12 months after SCI in 9/23 studies, International Association of Neurorestoratology (IANR) score in 1/23, neurologic level of injury (NLI) in 1/23, and summative or sequential motor/sensory scores in 2/23 studies.

Functionality status was assessed using functional ambulation category (FAC) in 1/23 studies, functional independence measure (FIM) score in 1/23, self-care ability in 1/23, and walking ability either after 6 or 12 months after SCI in 3/23 studies. Hospital/ICU utilization was evaluated using length of hospital and ICU stays in 3/23 studies, along with re-hospitalization frequency in 1/23 studies. Discharge destinations were dichotomized into home and non-home in 2/23 studies, while survival studies were measured with in-hospital, 1-year, and 7-year mortalities in 2/23 studies. Complications, as defined by the authors, were also documented in 2/23 studies. All these outcomes were subjects of prediction at a certain point in time after SCI. Neurologic status outcomes were utilized to recommend management strategies for the acute phase of SCI, like mean arterial pressure range in 1/23, acceptable time frame for MAP to be out of the recommended range in 1/23, and maximum time delay between SCI and surgical intervention before the hopes of recovery diminish in 1/23 studies. Image segmentation identified and characterized normal spinal cord, lesion, and radiomics, besides measuring lesion-specific findings, such as total length and volume of intraparenchymal hematoma. One study showed the capability of a cluster-based MLA to classify SCI patients into five sub-groups using a complex interaction between age, body mass index, NLI, energy of trauma mechanism, severity of SCI, length of stay in a hospital, and FIM score.

## Discussion

Our review shows artificial intelligence can have broad applications for patients with SCI. Of note, the studies’ overall quality of development and risk of bias are of high concern mainly due to the small number of cohorts the developers used both during MLA development and validation. This has also been noted by the group that developed PROBAST + AI [15] after reviewing multiple studies that used AI to develop predictive model within the health sector. To better understand the MLAs in the studies we reviewed, ChatGPT [14] was queried with the list of all specific MLAs to generate their family and the basis for the categorization under a particular family [14]. According to the response (Supplementary material S2), the six families differ in the data they process and the method of processing data. Deep Learning Models are data-driven models that use multiple layers of artificial neurons to learn representations like images. Classical Machine Learning Models include models that do not rely on deep learning, but rather statistical and decision-based methods. Ensemble Learning Models are combinations of multiple models to improve performance. Hybrid/Combined models integrate multiple modalities. AutoML-generated models are those that are built via automated machine learning pipelines. Unsupervised Learning Models are used when labels are not available. Ensemble model subtypes are included in both the Classical Machine Learning Family and the Ensemble Family because they combine multiple models with classical machine learning models as their foundation. However, Ensemble models are unique because they combine predictions of multiple models to reduce variance (e.g., bagging), bias (e.g., boosting), or both variance and bias (e.g., stacking) [14]. The various applications of MLAs in SCI include image segmentation to diagnose pathological features in MRI, predicting various outcomes, determining patient subgroups, and identifying key management guiding factors from the predicted outcomes and identified features. In practice, all these focus areas are interdependent, and drawing a solid line of separation is not possible. The discussion below is classified into each study’s focus areas and summarizes the prominent findings.

### Image segmentation

Medical image segmentation has gained broad interest for various specialties and sub-specialties of medicine [51]. In SCI, AI/ML has been used to detect and measure lesions in the spinal cord and identify a normal cord. According to the study by Naga Karthik et al. [42], the ML they used (SCIseg) achieved a mean dice score of 0.92 ± 0.07 (SD) and 0.61 ± 0.27 for spinal cord and spinal cord injury lesion segmentation, respectively, showing a high accuracy. Furthermore, both the manually- and SCIseg-identified lesion length, maximal axial damage ratio, and lesion volume were found to have a similar correlation to discharge pin prick, light touch, and lower extremity motor scores. The correlation between lesion character and discharge scores had statistical significance for lesion volume and insignificance for lesion length and maximal axial damage, for both manually and SCIseg identified lesions proving the accuracy of SCIseg. Further proving the accuracy of ML in image segmentation, Artha Wiguna et al. [49] showed models successfully identified the spinal cord and classified the severity of cervical SCI on T2-weighted MR images. They also noted satisfactory performance and feasibility to apply in clinical settings since the ROC-AUC value for ML classification models was 0.79, surpassing a human expert physician (ROC-AUC = 0.65) for MRI images from 50 random patients not previously used for training. Unfortunately, an average of 21.72 ± 18.21 mm lesion length difference was observed between AI and manual segmentation for the sagittal MRI showing the need to train and improve the performance of the AI used.

Another remarkable application is ML’s ability to successfully segment MRI, differentiating SCI and control patients, categorize injury severity, and localize lesions in images with artifacts from implants and instruments using radiomic feature analysis [44]. This was possible through an automated ML (H20 AutoML) that integrated outcomes of segmentations from various models, like random forest, GBM, XGBoost, neural networks, and more [44]. Gradient boosting machine (GBM) showed the best performance, except in the zone of injury classification using T1W MRI, where XGBoost was optimal. In situations where implant or instrument created artifact, T1 segmentation data were registered to the T2 space using spinal cord toolbox deep learning model’s registration module [52]. T1 and T2 segmentations were then integrated using the radial basis function algorithm. The integration segmentation leveraged complementary information, producing a robust result with ROC-AUC of 1.00, 0.99 and 0.98 for discriminating between healthy and Individuals with SCI, predicting injury severity and lesion zone classification, respectively. This is a unique function that was made possible because of the ML process.

### Prediction of outcome and management suggestions

The standard MAP value range that is considered standard (85–90 mmHg) to maintain within the first 5–7 days after SCI is based on level 4 evidence from a small center case series study [53, 54]. The recommendation has been widely adopted in clinical practice, but remains controversial due to limited evidence supporting pressure ranges and treatment duration [50, 55–57]. Unfortunately, generating a high-quality, authoritative result would not be feasible due to ethical considerations. Torres-Espin et al. [46] however, were able to process intra-op Q5 min MAP records from two centers in California leveraging AutoML’s high computational ability. They were able to identify the optimal MAP range that would result in AIS improvement (76- [104–117] mmHg) using ML to reduce data dimensionality while maintaining topology, k-NN clustering, LASSO feature reduction, and identifying the best logistic regression model for predicting AIS improvement. Using this finding as a foundation, Agarwal et al. [28] further identified that the most important factors for predicting AIS grade improvement were the amount of time the intraoperative MAP was in an extreme range, the brain and spinal injury center (BASIC) score from the admission MRI, and the average intra-op MAP. The authors [24, 28] also demonstrated that the ideal intra-op MAP range is 76–104 mmHg, 93 min was the total amount of time intra-op MAP could be above 104 mmHg or below 76 mmHg to have improved AIS at discharge, and the ideal intra-op average MAP should be between 80–96 mmHg as 100% of the patients with an average intra-op of 96.3 mmHg showed no improvement (same or worse) in AIS at discharge. All these findings would have been impossible to arrive at using conventional clinical trial models using human subjects for ethical and practical reasons. However, due to the complex and high computational capacity of ML algorithms, we can fill gaps, as demonstrated in such studies, using prospectively collected and processed human data, without interfering with SCI care in keeping with local practice and guidelines. Agarwal et al. [28] also identified phenylephrine as a safer drug compared to dopamine for MAP augmentation since it had significantly lower rates of tachycardia and atrial fibrillation/flutter associated with its use.

Another study by Goulet et. al. [35] assessed the influence of surgical timing on neurological recovery to identify objective surgical timing cutoffs associated with better neurological recovery. Using classification and regression tree (CART) analysis in patients that sustained a motor-complete (AIS A and B) SCI secondary to a thoracolumbar injury, they found that the proportion of patients who improved by at least one AIS grade was higher in the group undergoing early surgery within 25.7 h of SCI (46 vs 0%). The proportion of patients that improved by at least one NLI was also higher in the group undergoing early surgery within 21.5 h of SCI (71 vs 18%) and 25% of the AIS grade A patients undergoing early surgery within 25.6 h improved 10 motor score points or more as compared with 0% in the other group. Goulet et al. [35] also identified that while the initial AIS grade was critical in motor score improvement, surgical delay remained predictive for AIS improvement in AIS grade A patients, thus identifying the complex interaction between injury features and surgical intervention in determining recovery.

### Prediction of outcomes

SCI is known for its devastating impact and creating irrecoverable disabilities that affect victims, family members, and society in general [1, 2, 58]. For this reason, it has been a significant center of research, especially around recovery after injury [59, 60]. Not surprisingly, with the availability of large data sets and the complex computational ability of artificial intelligence, the prediction of the various facets of outcomes after SCI has gained more attention in the application of AI in SCI. A majority of the studies tried to capture the multi-level interaction of various clinical variables to determine neurologic and functional recovery in terms of AIS improvement [28, 31, 35–37, 40, 43, 45, 46], IANR score [48], functional ambulation category (FAC) [47], walking ability [31, 32, 41], Functional Independence Measure (FIM) scores [41], Spinal Cord Independence Measure (SCIM) score [12], motor and sensory score improvement [31, 35] and NLI level change [35]. The rationale for these predictions, according to the various authors, is to facilitate decision-making regarding patient care and counseling the patient, family members, and caregivers. There were also significant results that were identified regarding controversial and unconfirmed areas of decision-making during the acute care of Individuals with SCI as discussed below.

#### Neurologic status prediction

Shimzu et al. [45], Inoue et al. [36], and Okimatsu et al. [43] focused on identifying predictors of AIS improvement at discharge [45], 1 month [43], and 6 months [36, 45] in patients with cervical spinal cord injury. AIS was evaluated as five categories (A, B, C, D, and E) at the designated time by Shimzu et al. and Okimatsu et al., while it was dichotomized as A/B/C or D/E by Inoue et al. A common finding in these studies is that the prominent predictors of AIS outcomes are initial AIS scores, MRI-based lesion characters either identified by deep learning models or identified on MRI as the presence of intramedullary hemorrhage, the lesion length, or BASIC score (specifically BASIC 3 and 4 which are at the worst end). Shimzu et al. additionally identified motor index score in lower extremities and HbA1C status as important predictors. The explanation for the importance of HbA1C was that hyperglycemia increases the inflammatory response by over-activating NF-kB in microglial cells, which exacerbates secondary injury, thus unfavorably affecting ASIA motor score and recovery rate. While Maximum Canal Compression (MCC) and Maximum Spinal Cord Compression (MSCC) were important predictors according to their findings, they acknowledged that intramedullary findings might be more important than these MRI biomarkers, especially considering other studies [61, 62]. In addition to these findings, Okimatsu et al. [43] stated that a combination of deep learning radiomics that utilized MRI findings (within 24 h of SCI) and a random forest model that utilized clinical data like initial AIS was more accurate (0.714) in predicting AIS after 1 month of SCI compared to each model’s independent performance. The other studies [31, 37, 40, 48] that focused on all vertebral levels of SCI had similar findings regarding initial AIS, motor score, completeness of injury, and delay of care, predicting AIS status at a similar time after SCI. Regarding the prediction of neurologic improvement, in all the studies, ML accuracy and performance metrics improve when ML models are combined. A peculiar finding by Kapoor et al. [37] was the identification of age as a weak predictor of AIS change. While age played some role, there were several other features, such as sex and race, that were also around the same level of absolute mean importance. However, age was of disproportionately higher importance for AIS grade C predictions. Overall, the findings generally agree with studies that use conventional statistical methods [54, 61, 63–65] while ML clearly identified complex interactions among the predictors in determining outcomes.

#### Functional outcome prediction and patient sub-grouping

The other application of AI/ML was determining functionality after a set time in patients with SCI. Because functional recovery is highly dependent on neurologic recovery, the predictors identified using ML were fairly identical to the predictors of AIS status. However, when assessing functionality, the outcomes were based on scoring systems that use continuous measurements. Yoo et al. [47] developed an MLA to predict the Functional Ambulation Category (FAC) score at discharge. The most significant predictors of FAC at discharge were the initial FAC, lower extremity motor scores (bilateral hip flexor, bilateral ankle dorsiflexor, and left knee extensor), NLI, age, and periods of acute care. The patients with an initial FAC level below 2 with ankle dorsiflexor weakness and NLI in the cervical or thoracic region showed the worst outcome (FAC 0). Zhang et al. [48] used the International Association of Neurorestoratology score to dichotomize patients with≥ 37 or <37 points to define good and bad prognoses, respectively. The MLA generated predictors that determined which category the patients would be in 6 months after cervical SCI. Furthermore, a combined model integrating radiomics and clinical features demonstrated excellent prediction performance, with an ROC AUC of 1.000 in the training set, 0.952 in the testing set, and 0.815 in the validation set, showing age, diabetes, admission AIS, and treatment were independent clinical risk factors. Facchinelo et al. [12] used the SCIM score to measure functionality within the first year after SCI. The initial AIS grade was the most significant predictor, followed by Injury Severity Score (ISS), surgery delay, NLI, pressure ulcer, age, trauma mechanism, and urinary tract infection for SCIM.

DeVries et al. [32] compared a cluster analysis-based MLA with established classic prediction models [66, 67] to determine walking ability at discharge and 1 year after SCI. The locomotion portion of FIM scores 6 and 7 defined the walking group (functional walking), while scores 1–5 defined the non-walking group (non-functional ambulation). The patients able to walk at discharge or follow up were older, with more time elapsing before examination and with more AIS C and D. The MLA successfully identified this pattern, and no statistical differences existed between MLA and the previously validated models when comparing ROC-AUCs, despite the greater amount of neurological data used by the MLA. Maki et al. [41] on the other hand, analyzed predictors of walking ability the same way as De Vries et al., using FAC, and further analyzed predictors of a secondary outcome, i.e., motor section for FIM. The MLA identified initial ASIA motor score, age, initial total FIM score, number of days from injury to admission, and NLI as factors that strongly influence the model’s output for both FIM ambulation and motor score predictors. The findings by Yoo et al., Zhang et al., Facchinelo et al., De Vries et al., and Maki et al., despite using various, albeit overlapping, measures of functionality, all concluded that initial neurologic, motor, and functional status mattered for the final outcomes. In addition, these studies determined that age, delay in treatment/intervention, and NLI were involved in predicting functionality.

The need to identify and further understand the heterogeneity in recovery and prognosis patterns after SCI motivated the development of an unsupervised patient clustering MLA model by Basiratzadeh et al. [30]. The authors emphasized knowing the individual variables gives limited information about the complex interaction among other variables to predict prognosis. They also mention that while the AIS grade itself might be the most important indicator for the prediction of recovery, other clinical factors such as age, injury characteristics, and functional measures have also been reported as significant prognostic variables. With this aim, the MLA they developed recognized 5 subgroups (Subgroups 1 - 5) of Individuals with SCI based on demographics (age, BMI), functional capacity, injury characteristics at admission (Motor score, NLI and Mechanism of Injury) and outcome variables (LOS, FIM and motor score at discharge).

#### Prediction of hospital utilization

The other focus of prediction for Individuals with SCI is hospital utilization in terms of Length of Stay (LOS), both total and ICU stay, and re-hospitalization. Fan et al. [34] used a histogram of stay to define prolonged LOS-ICU as >7 days (26% population), and a prolonged LOS-hospital as >14 days (26% population). Predictors were identified using an ensemble ML model and multiple input data types that included demographics, various and extensive laboratory parameters, treatment information, including mechanical ventilation, and extra information like source of admission, LOS before ICU admission, and care unit. The top five important features for LOS-ICU were mechanical ventilation, the total number of diagnoses, RBC, hemoglobin, and magnesium. The top five important features for LOS-hospital were: mechanical ventilation, the total number of diagnoses, LOS before ICU admission, bicarbonate, and chloride. In brief, patients with mechanical ventilation and more diagnoses were most likely to incur prolonged ICU and hospital stays. The ROC AUC for these predictions ranged from 0.796–0.802, depending on the ensemble ML type. In contrast, Karaback et al. [38] stated LOS was prominently predicted by the American College of Surgeons (ACS) verification level of the hospital, systolic BP, race, age, and total Glasgow Coma Scale (GCS). Systolic BP, the hospital’s ACS verification level, pulse-oximetry, and pulse rate were the top four predictors of prolonged LOS in the ICU. Major complications were prominently predicted by pulse oximetry, supplemental oxygen, total GCS, respiratory rate, respiratory assistance, GCS-verbal, and temperature. In this study, the cumulative rather than individual predictive effects of the other 75 features/variables were more powerful in determining prolonged LOS (both hospital and ICU) and major complications.

Aly et al. [29] focused on predicting rehospitalization using a tree-model MLA (random forest) and discovered the most important predictors are higher FIM motor scores, ASIA sensory and motor scores, deep anal pressure, higher educational degrees, occupation, having private insurance, alcohol drinking rate, higher BMI, the time from injury to admission, and family income. They also identified contributors to prolonged LOS after re-hospitalizations, which included pressure injuries, urinary tract infections, low FIM, AIS, older age, and associated injuries. Their aim was to help clinicians identify high-risk patients before discharge to optimize treatment as needed on a timely basis.

#### Prediction of discharge destination and survival/mortality

Fallah et al. [33] used multiple ML techniques to design and validate a new Spinal Cord Injury Risk Score (SCIRS) that can predict mortality based on demographics and injury mechanics, which was compared with the Injury Severity Score (ISS). The score ranges between 0 and 28, with higher numbers indicating a higher probability of dying in the hospital, within one year, or overall. The variables utilized to create the new SCIRS were age, AIS, NLI, AO spine injury morphology, and Abbreviated ISS (AISS) for face, neck, thorax, abdomen, lower extremity, and upper extremity. AISS for spine was excluded because of the use of NLI and AIS to assess the injury severity of the spine. Using machine learning, the authors identified the weight of several variables in determining in-hospital, 1-year mortality, and 7-year mortality. Using the weight identified for each variable, they assigned scores to the variables; the sum of the scores yields the SCIRS, which determines the probability of mortality within the time frames with better performance metrics than the ISS. Karaback et al. [38] also assessed predictors of mortality and discharge destination using similar inputs as Aly et al. The top 3 predictors of in-hospital mortality were total GCS, age, and the verbal component of GCS; however, increasing age had a strong association with non-home discharges. The cumulative rather than individual predictive effects of the other 75 features were more powerful in determining discharge destination and in-hospital mortality.

Finally, Kato et al. [39] identified predictors of discharge destination using admission and discharge time features using classification and regression trees. For admission features, ≥5 points for mobility score of SCIM, age (≥or <74 years), and upper extremity score (≥or <23 points) were the top three predictors. A total SCIM score of 40 was the only discharge feature that was found to be impactful in determining discharge destination. This total SCIM was achieved by those having NLI C6 and below, implying that patients with an NLI above 6 have a lower chance of being discharged to home.

## Limitations

This review has several limitations. First, the pace at which AI and ML models are being developed and applied is rapid. With the continuous advancement of the field, more models and applications are expected to emerge, quickly rendering the information available now as outdated. We encourage other researchers to conduct frequent reviews to address this issue. Importantly, despite the general idea of the use of AI in SCI as depicted in this study, the models are poorly developed and validated, possibly exaggerating their accuracy or applicability, especially in sensitive issues like long term outcomes and survival in SCI. The studies are also retrospective in nature and their level of evidence is low. The other limitation is that we focused on the application of AI in acute and sub-acute time frames after SCI, with no focus on long-term rehabilitation strategies that extensively use AI. One example is the application of AI in brain computer interface to stimulate the spinal cord and the integration of robotics into the realm of SCI. We intentionally did not focus on those areas due to the vastness of the field and to contain the scope of this review. Another limitation is that our focus on the application of AI in traumatic SCI provides a general idea of the current state of knowledge but does not offer specific information about other non-traumatic SCI scenarios. Therefore, given these limitations, further review is needed to gain a full picture of the application of AI to Individuals with SCI. In addition, the clinical applicability of MLAs is not yet endorsed by institutions due to a lack of prospective studies that can build the trust of medical professionals. Despite these limitations, however, the authors believe that there is enough to digest in the applications already covered in this review, and future developments should help overcome these limitations.

## Conclusion

Artificial intelligence can be leveraged to assist a personalized approach in diagnosing SCI and predict various outcomes after SCI, including neurologic improvement. Furthermore, AI can help predict functionality indicators (e.g., walking, self-care, and independence), hospital utilization (e.g., readmissions and prolonged lengths of stay), discharge destination, and mortality at different time frames. AI can also be used to predict complications and suggest various management strategies, like specific MAP target goals and the time point of surgical intervention. The various performance metrics show high accuracy, sensitivity, specificity, and precision that can surpass human capacity. The excellent performance was due to the combination of various models in the function needed. These functions can be combined to assist in delivering personalized and high-quality care to Individuals with SCI and assist in clinical judgement.

Level of Evidence - III

## Supplementary information

Supplementary Material S1

Supplementary Table S2

Supplementary Material S3

## Data Availability

Data for this study is found within the reviewed studies which are cited.
